# Fault analysis and performance improvement of grid-connected doubly fed induction generator through an enhanced crowbar protection scheme

**DOI:** 10.1371/journal.pone.0327802

**Published:** 2025-07-29

**Authors:** Rameez Akbar Talani, Ghulam Sarwar Kaloi, Aamir Ali, Ghulam Abbas, Ahmed Emara, Ezzeddine Touti

**Affiliations:** 1 Department of Electrical Engineering, Quaid-e-Awam University of Engineering Science and Technology, Nawabshah, Sindh, Pakistan; 2 School of Electrical Engineering, Southeast University, Nanjing, China; 3 Department of Electrical Engineering, University of Business and Technology, Jeddah, Saudi Arabia; 4 Department of Engineering Mathematics, and Physics, Faculty of Engineering, Alexandria University, Alexandria, Egypt; 5 Center for Scientific Research and Entrepreneurship, Northern Border University, Arar, Saudi Arabia; SRM-RI: SRM Institute of Science and Technology (Deemed to be University) Research Kattankulathur, INDIA

## Abstract

The main problem associated with a doubly fed induction generator (DFIG) during fault is large inrush currents induced in rotor winding, which has detrimental effects on the machine’s AC excitation converter. A simple conventional resistance inclusion (crowbar) is employed with a PI controller to protect a DFIG from transient current, but it is observed that this method is not enough to keep transient over-current to an admissible level. In this paper, an effective current limiting technique along with reactive power control is proposed in order to maintain stability, reduce transient current surge to an acceptable level, and enhance the Fault Ride Through capacity of DFIG. The proposed dynamic control technique not only limits the fault current during voltage dip to a permissible level but also controls the reactive power during fault. The behavior of a proposed technique is analyzed by introducing unsymmetrical faults in the MATLAB-based model of DFIG. An enhanced crowbar-based fault ride-through is employed for the rotor side controller to limit inrush current and control reactive power.

## 1. Introduction

Recent advancements in intelligent control and optimization of power generation systems have significantly improved the efficiency and reliability of renewable energy systems. An intelligent control system for power generation using DSPACE was proposed in [1] enhancing operational performance and adaptability. Similarly, bearing fault diagnosis in direct torque control systems was explored by integrating neural networks and fuzzy logic, offering a comprehensive analysis of fault detection techniques [2]. In wind energy systems, authors presented a robust control system for DFIG-based Wind Energy Conversion Systems (WECS) with energy storage, demonstrating its effectiveness in real-world wind conditions [3]. The performance of grid-connected photovoltaic-electric vehicle charging stations using model predictive control was improved, addressing dynamic power management in real-time [4]. Battery capacity estimation has also been enhanced, who applied the Bald Eagle Search algorithm to improve accuracy in energy storage systems [5]. The optimization of Maximum Power Point Tracking (MPPT) controllers for wind energy conversion systems has been studied by comparing various strategies to improve system performance [6]. Furthermore, authors investigated state-of-health estimation for lithium-ion batteries in electric vehicles, using gradient-based optimization techniques to improve battery life estimation accuracy [7]. Loulijat et al. focused on enhancing fault ride-through capacity in DFIG-based wind power systems using adaptive backstepping control, contributing to the stability and robustness of wind turbines under grid disturbances [8]. Finally, Hadoune et al. optimized direct power control for DFIG-based WECS using the super-twisting algorithm, underlining the importance of advanced control techniques in real wind conditions [9].

With the rapid increase in demand for green energy, the reliance on renewable power generation increases. Among renewable power generation, wind energy is a clean source of energy. Doubly fed induction generator (DFIG) is the most widely used wind turbine for renewable power generation because of its high efficiency and ability to operate at variable speeds [10]. In a DFIG-based wind energy conversion system, the stator is directly linked to the grid, whereas the rotor is indirectly connected through the two back-to-back connected converters named rotor side converter or Machine side converter (RSC) and grid side converter (GSC) [11]. The study presents a control strategy that enhances the fault ride-through capability of DFIG-based wind turbines. By integrating Fuzzy Logic Control (FLC) for fault detection and Salp Swarm Optimization for parameter tuning, it improves power quality and system stability during faults [12]. The RSC primarily controls the rotor’s active and reactive power, while the GSC ensures the stability of the DC-link voltage and regulates power exchange between machine and the grid [13]. This configuration offers flexibility in controlling power output and enhances the overall system efficiency [14]. However, grid-connected DFIG exhibits dynamic instability during grid faults, which causes the power output fluctuation and system failure. Advanced control strategies have been developed to improve the fault ride-through (FRT) capability of DFIG-based wind turbines, particularly during any grid faults [15], With rapid increase in use of Doubly Fed Induction Generator-based Wind Energy Conversion Systems (DFIG-WECSs), maximizing power extraction from win and improving fault ride-through (FRT) capability are critical. The approach discussed in paper [16] proposes a dual-mode control scheme for normal and fault conditions to optimize system performance.

There are three main control approaches to achieve the fault-ride through (FRT) capacity of DFIG: first is employing the control strategy of active and reactive power control, second is using protective devices such as a crowbar, and third is controlling the parameters of back-to-back connected controllers [17]. The simultaneous control of active and reactive power is established with the inclusion of DC-bus tension, which is discussed in [18]. The unknown parameters are estimated by applying the parameter adaption algorithm technique. A real-time power control approach by integrating an experimental setup using the Dspace DS1104 board is proposed in [19]. This approach controls the voltage and frequency of the DFIG stator within the permissible limit during fault. A hierarchical fault-tolerant approach is introduced in [20] that consists of maximum power point tracking (MPPT) and pitch angle control (PAC). This hierarchical strategy controls the system parameters during short-circuit faults, wind variation, and undesirable surges in DC voltage. A control technique is proposed to enhance the FRT meeting the grid code [21], which consists of superconducting magnetic energy storage and a series transformer incorporated together. During normal power variation caused by wind speed variation, GSC is controlled, and under severe fault conditions, a protective circuit operates to limit the fault current. In [22], a combination of two active crowbars installed at the DC-link side and RSC side is proposed. These combinations of crowbars control the stator and rotor transient current and DC-link voltage during symmetrical and unsymmetrical faults. Two crowbar-based impedance circuits are used in [23]. The proposed strategy consists of a series of R-L impedance circuit and parallel R-L impedance circuit applied together to protect the RSC and DC-link capacitor against the fault currents, respectively. Similarly, in [24], the crowbar modification technique is employed by including an RpLp circuit integrated in parallel with the traditional crowbar. This parallel connection of the RpLp circuit enables the DFIG to exhibit the attributes of both a squirrel cage induction generator (SCIG) and DFIG in hybrid mode, which improves the system’s stability against symmetrical faults. Naderi et al. introduced a DC chopper to protect the DC-link capacitor, limit fault current, and control the DC link voltage, thus improving FRT capability [25]. In another study [26], an improved FRT is proposed by considering the resonance and different parameters when designing the capacitance using an RC-crowbar design. This strategy improves the stator voltage recovery and controls the GSC parameters during the fault period. The main problem associated with the crowbar is the inappropriate time during activation and deactivation between and after the fault, which may result in the recurrence of the inrush current. In [27], an algorithm is proposed to prevent this recurrence of inrush current during crowbar operation. Similarly, in [28], the inappropriate switching time of the crowbar is improved by using a fuzzy logic controller under symmetrical fault, hence improving the FRT capability of DFIG. The technique proposed in [29] solves the inappropriate activation of crowbar timing in two stages by analyzing the fault characteristics at the RSC control stage and controlling the fault at the crowbar activation stage. A heuristic technique is proposed in [30] to control suitable crowbar resistance. Once the optimal crowbar value is obtained, the linear quadratic regulator is employed in the system. The function of a linear quadratic regulator is to ensure that FRT meets the grid code requirements. Three three-stage dynamic equivalent methods of a DIFG-based wind farm are discussed in [31]. In this method, different factors of dynamic stages are considered, which include crowbar identification, voltage differences, and wind speed differences of multiple DFIGs connected in wind farms. The purpose of this study is to enhance the LVRT process and improve the overall wind farm stability. A modified crowbar circuit design of a grid-connected DFIG is introduced in [33]. The new control circuit shows an improved crowbar control strategy in an unsymmetrical fault condition. In [33], multiple three topologies of a crowbar are designed, and their effects on voltage, current, and torques are analyzed. The proposed fault protection scheme increases the FRT capability of DFIG and reduces the detrimental effects of fault current on RSC. In the paper [34], an active crowbar protection scheme is proposed to improve the FRT capability of DFIG. Unlike the conventional crowbar, which includes only a resistor, the proposed design includes a capacitor connected in series with the resistor of the crowbar. The purpose of a capacitor is to reduce the rotor current ripples and protect the RSC and DC-link capacitor. Several studies have focused on improving the fault ride-through (FRT) capability of Doubly Fed Induction Generator-based Wind Energy Conversion Systems (DFIG-WECSs). An improved crowbar control circuit has been proposed to enhance system stability during low-voltage ride-through (LVRT) [35], while modeling and simulation of crowbar protection techniques under symmetrical fault conditions have been explored [36]. Short circuit faults have been analyzed, with the presentation of an active crowbar protection circuit to improve power quality during faults [37]. Grid frequency and amplitude control using DFIGs in smart grids to enhance grid stability has been investigated [38]. Enhancement of crowbar hardware design for improved FRT in DFIG-based systems has also been a key area of focus [39], with further research into transient behavior and fault current assessment with crowbar hardware [40]. The study in [41] analyzes Yemen’s energy system, focusing on its challenges, including energy efficiency, institutional capacity, and grid losses. The study in [42] evaluates Yemen’s energy system, addressing its inefficiencies, high losses, and potential for decentralized small-scale power generation solutions.

From the available literature, the three main drawbacks of the crowbar are observed; firstly, when the fault occurs, it turns off the RSC; as a result, DFIG starts working as a squirrel cage induction machine, which takes up reactive power from the grid, and this high absorption of reactive power does not meet grid code requirement, secondly when the more severe fault occurs the conventional technique is inadequate in handling the large transients in rotor current and thirdly when RSC turns on after fault and crowbar deactivate results in high inrush currents and transient oscillations which may damage the RSC. In the literature discussed, many techniques are proposed to overcome these problems separately, either to control the proper switching of the crowbar to reduce the fault severity or to control reactive power during the fault, and very little literature is available regarding collaborative operation to solve major drawbacks together. This paper simultaneously covers the major drawbacks of the crowbar-based FRT approach and proposes an enhanced crowbar technique, which is active impedance-based. When the crowbar is activated during the fault, the optimal value of the crowbar is taken to limit the fault current amplitude; simultaneously, the reactive power is controlled through the regulator, which further increases the system’s stability. During post-fault scenario after RSC activation causes the large inrush current; active impedance used at the controller output limits the inrush current after the fault. The behavior of the proposed model is analyzed under single-phase and more severe two-phase asymmetrical faults.

**Fig 1:**
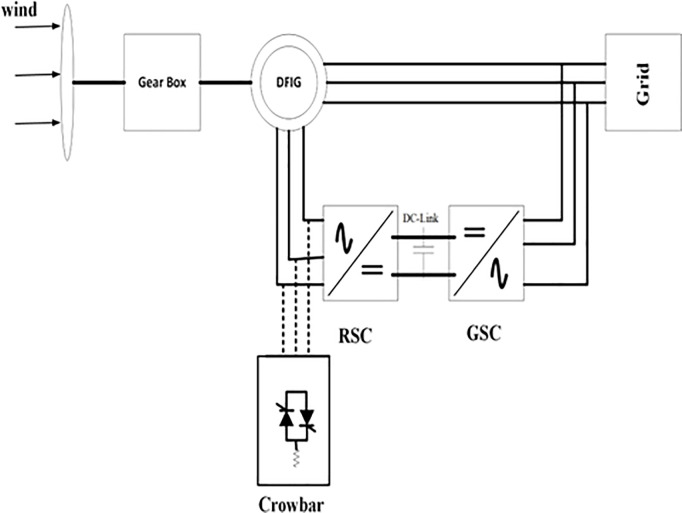
0 basic diagram of DFIG-based wind turbine with a crowbar protection.

The basic operations of DFIG based-wind turbine with conventional crowbar protection is given in Fig 1. It shows the basic components of DFIG-WT which consists of the rotor side converter connected to DFIG, grid side converter connected to grid, gearbox at the shaft of rotor, both converters are connected via DC bus and RL filter, and crowbar positioning in the RSC side of DFIG, during fault this crowbar is used to bypass the fault current and deactivates the RSC.

The rest of the paper is followed by section 2, describing the dynamic modeling of the rotor and stator. Section 3 introduces the proposed approach. Section 4 gives the results and discussion obtained from simulations and a quantitative comparison of obtained results with literature, respectively, while the conclusion of the study is drawn in section 5.

## 2. Dynamic modeling of rotor and stator

This portion deals with the dynamic modeling of the machine to calculate the faults and minimize the transient currents and voltage dip in DFIG. The voltages and flux induced in rotor and stator side of machine are determined by enhancing direct-axis (d-axis) and quadrature-axis (q-axis) DC components in a synchronous reference frame.


$$V_{ds}=R_{s}i_{ds}-\;\omega_{s}\psi_{ds}+\;\frac{d\psi_{ds}}{dt}\;$$ (1a)

$$V_{qs}=R_{s}i_{qs}+\;\omega_{s}\psi_{qs}+\;\frac{d\psi_{qs}}{dt}\;$$ (1b)

$$V_{dr}=R_{r}i_{dr}-\;s\omega_{s}\psi_{dr}+\;\frac{d\psi_{dr}}{dt}$$ (2a)

$$V_{qr}=R_{r}i_{qr}+\;s\omega_{s}\psi_{qr}+\;\frac{d\psi_{qr}}{dt}$$ (2b)

$$\psi_{ds}={(L}_{s}+L_{m})i_{ds}+L_{m}i_{dr}$$ (3a)

$$\psi_{qs}={(L}_{s}+L_{m})i_{qs}+L_{m}i_{qr}$$ (3b)

$$\psi_{dr}=L_{m}i_{ds}+{(L}_{r}+L_{m})i_{dr}$$ (4a)

$$\psi_{qr}=L_{m}i_{qs}+{(L}_{r}+L_{m})i_{qr}$$ (4b)

Where $V_{ds}$,$\;V_{qs},V_{dr},\;V_{qr}$ represents stator and rotor voltages in d-q axes, whereas $\psi_{ds},\psi_{qs},\psi_{dr},\psi_{qr}\;$ shows magnetizing fluxes of the stator and rotor. $\omega_{s}$, $R_{r},\;R_{s}\;and\;s$ represents the synchronous speed, rotor resistances, stator resistances, and slip, respectively [32,33].

Equations 5a and 5b are obtained by putting the value of equations 4a and 4b into equations 2a and 2b:


$$v_{dr}=R_{r}i_{dr}-\;s\omega_{s}\left(\left(L_{r}+L_{m}\right)i_{dr}+L_{m}i_{ds}\right)+\left(L_{r}+L_{m}\right)\frac{{di}_{dr}}{dt}+L_{m}\frac{{di}_{ds}}{dt}$$ (5a)

$$v_{qr}=R_{r}i_{qr}+\;s\omega_{s}\left(\left(L_{r}+L_{m}\right)i_{qr}+L_{m}i_{qs}\right)+{(L}_{r}+L_{m})\frac{{di}_{qr}}{dt}\;+L_{m}\frac{{di}_{qs}}{dt}\;$$ (5b)

Stator current in equations 6a and 6b are obtained from equations 3a and 3b:


$$i_{ds}=\left(\frac{1}{L_{m}+L_{s}}\right)\psi_{ds}-(\frac{L_{m}}{L_{s}+L_{m}})i_{dr}$$ (6a)

$$i_{qs}=\left(\frac{1}{L_{m}+L_{s}}\right)\psi_{qs}-(\frac{L_{m}}{L_{s}+L_{m}})i_{qr}$$ (6b)

Putting the values of equation 5a, 5b and 6a, 6b into equation 4a,4b we get an equation 7a,7b:


$$\begin{array}{cc} V_{dr}= & {\left(R_{r}+R_{s}\frac{{L_{m}}^{2}}{{(L}_{s}+L_{m})²}\right)i}_{dr}-s\omega_{s}\left(\left(L_{m}+L_{r}\right)-\frac{L_{m}^{2}}{L_{s}^{2}}\right)i_{dr}+\left(L_{m}+L_{r}-\frac{L_{m}^{2}}{{\left(L_{m}+L_{s}\right)}^{2}}\right)i_{dr}\\ & +\;\left(\frac{L_{m}}{{(L}_{m}+L_{s})}\right)\left(\left(\frac{R_{s}}{{(L}_{m}+L_{s})}\right)\psi_{ds}+\omega_{s}\psi_{ds}-\;s\omega_{s}\psi_{dr}\right) \end{array}$$ (7a)

$$\begin{array}{cc} V_{qr}= & \left(R_{r}+R_{s}\frac{{L_{m}}^{2}}{{(L}_{s}+L_{m})²}\right)i_{qr}-s\omega_{s}\left(\left(L_{m}+L_{r}\right)-\frac{L_{m}^{2}}{L_{s}^{2}}\right)i_{qr}+\left(L_{m}+L_{r}-\frac{L_{m}^{2}}{{\left(L_{m}+L_{s}\right)}^{2}}\right)i_{qr}\\ & +\;\left(\frac{L_{m}}{{(L}_{m}+L_{s})}\right)\left(\left(\frac{R_{s}}{{(L}_{m}+L_{s})}\right)\psi_{qs}-\omega_{s}\psi_{qs}+\;s\omega_{s}\psi_{qr}\right) \end{array}$$ (7b)

The stator of DFIG is directly connected to the grid, during grid fault this effects the stator parameters and causes stator voltage dip. This change in stator parameters and stator flux variation effects the back emf induced in the rotor. Therefore it is necessary to calculate the stator flux value. Equation 8a and 8b represents the rotor back emf. The calculation of back emf helps to find the transient values of rotor current during fault. Equations 8a and 8b shows that any change in stator dynamics can effects rotor dynamics:


$$e_{d}=\frac{L_{m}}{{(L}_{s}+L_{m})}\left(v_{dr}+\omega_{s}\psi_{ds}-\;s\omega_{s}\psi_{ds}\;\right)$$ (8a)

$$e_{q}=\frac{L_{m}}{{(L}_{s}+L_{m})}\left(v_{qr}-\omega_{s}\psi_{qs}+\;s\omega_{s}\psi_{qs}\right)$$ (8b)

Rotor d-q axes current shown in equations 9a and 9b are obtained by isolating rotor current:


$$i_{dr}=\left(\frac{1}{{(L}_{m}+L_{r})}\right)\psi_{dr}-\frac{L_{m}}{{(L}_{m}+L_{r})}i_{ds}$$ (9a)

$$i_{qr}=\left(\frac{1}{{(L}_{m}+L_{r})}\right)\psi_{qr}-\frac{L_{m}}{{(L}_{m}+L_{r})}i_{qs}$$ (9b)

Equation 10a and 10b is obtain by putting equation 5a, 5b into equation 3a, 3b:


$$\psi_{ds}=\left[{(L}_{m}+L_{s})-\;\left(\frac{L_{m}^{2}}{{(L}_{m}+L_{r})}\right)\right]i_{ds}+\left(\frac{L_{m}}{{(L}_{m}+L_{r})}\right)\psi_{dr}$$ (10a)

$$\psi_{qs}=\left[{(L}_{m}+L_{s})-\;\left(\frac{L_{m}^{2}}{{(L}_{m}+L_{r})}\right)\right]i_{qs}+\;\left(\frac{L_{m}}{{(L}_{m}+L_{r})}\right)\psi_{qr}$$ (10b)

The dynamic modeling of stator flux linkage is obtained from above equations, where transient reactance can be represented as:


$$X^{’}=\omega_{s}\left(\left(L_{m}+L_{s}\right)-\frac{L_{m}^{2}}{{(L}_{m}+L_{r})}\right)$$

Equations 11a and 11b are obtained by putting the values of transient reactance X` in equations 1a, 1b and by avoiding flux linkages:


$$V_{ds}=R_{s}i_{ds}-\;X^{’}i_{ds}+e_{d}$$ (11a)

$$V_{qs}=R_{s}i_{qs}+\;X^{’}i_{qs}-e_{q}$$ (11b)

Equation 11a and 11b represents the dynamic modeling of stator voltage. In order to find the source voltage, which represents transient behavior, equations 9a and 9b are incorporated into equations 2a and 2b to get equations 12a and 12b [33]:


$$e_{d}^{\cdot }=\;\frac{R_{r}}{L_{r}^{`}}e_{q}+\;\frac{R_{r}}{L_{r}^{`}}\left(\frac{L_{m}^{2}}{L_{r}^{`}}\right)i_{ds}+\;s\omega_{s}e_{d}-\frac{L_{m}}{\left(L_{r}^{`}\right)}V_{dr}$$ (12a)

$$e_{q}^{\cdot }=\;\frac{R_{r}}{L_{r}^{`}}e_{q}+\;\frac{R_{r}}{L_{r}^{`}}\left(-\frac{L_{m}^{2}}{L_{r}^{`}}\right)i_{qs}-\;s\omega_{s}e_{q}+\frac{L_{m}}{\left(L_{r}^{`}\right)}V_{qr}$$ (12b)

Where $L_{r}^{`}$ shows the rotor transient inductance. Putting the values of stator current from equation 6a, 6b in equation 5a, 5b and simplifying the equation we get 13a, 13b:


$$V_{dr}=R_{r}i_{dr}+\left(\frac{-L_{m}^{.}}{L_{m}+L_{s}}+L_{m}+L_{r}\right){(i}_{dr})\left(-s\omega_{s}\right)+\left(\frac{L_{m}^{.}}{L_{m}+L_{s}}+L_{m}+L_{r}\right)\frac{{di}_{dr}}{dt}\;-\frac{s\omega_{s}L_{m}}{{(L}_{s}+L_{m})}\psi_{ds}$$ (13a)

$$V_{qr}=R_{r}i_{qr}+\left(\frac{-L_{m}^{.}}{L_{m}+L_{s}}+L_{m}+L_{r}\right){(i}_{qr})\left(s\omega_{s}\right)+\left(\frac{L_{m}^{.}}{L_{m}+L_{s}}+L_{m}+L_{r}\right)\frac{{di}_{qr}}{dt}+\frac{s\omega_{s}L_{m}}{{(L}_{s}+L_{m})}\psi_{qs}$$ (13b)

Equations 14a, 4b, and 15 show dynamic rotor voltage and stator flux:


$$V_{dr}=({R_{r}^{`}}_{.}{)(i}_{dr})+(L_{r}^{`}){(i}_{dr})+\left(\omega_{s})(L_{r}^{`}\right)\left(i_{dr}\right)-\left(\frac{L_{m}^{.}}{L_{s}}\right)\left(\frac{s\omega_{s}L_{m}}{{(L}_{s}+L_{m})}\right)$$ (14a)

$$V_{qr}=({R_{r}^{`}}_{.}{)(i}_{qr})+(L_{r}^{`}){(i}_{qr})+\left(\omega_{s})(L_{r}^{`}\right)\left(i_{qr}\right)+\left(\frac{L_{m}^{.}}{L_{s}}\right)\left(\frac{s\omega_{s}L_{m}}{{(L}_{s}+L_{m})}\right)$$ (14b)

$$\psi_{sdq}=\left(\psi_{sdq2}+(\psi_{sdq}-\psi_{sdq2})\;{e^{\sigma t}e}^{j\omega t}\right)$$ (15)

Where $R_{r}^{`}$and $L_{r}^{`}$ are the fault resistance and inductance of rotor current dynamics [34]. Equation 15 shows a change of stator flux during the fault, and σ is the stator flux damping. Equations 16a, 16b, and 17a, 17b show the rotor dynamics after crowbar activates and deactivates:


$$0=({R_{r}^{`}+R}_{crow}{)(i}_{dr})+(L_{r}^{`}){(i}_{dr})+\left(\omega_{s})(L_{r}^{`}\right)\left(i_{dr}\right)+E_{d}$$ (16a)

$$0=({R_{r}^{`}+R}_{crow}{)(i}_{qr})+(L_{r}^{`}){(i}_{qr})+\left(\omega_{s})(L_{r}^{`}\right)\left(i_{qr}\right)+E_{q}\;$$ (16b)

$$E_{d}=\frac{L_{m}^{.}}{L_{s}}\left(\psi_{sd2}-(\psi_{sd}-\psi_{sd2})\;{e^{\sigma t}e}^{j\omega t}\right)$$ (17a)

$$E_{q}=\frac{L_{m}^{.}}{L_{s}}\left(\psi_{sq2}-(\psi_{sq}-\psi_{sq2})\;{e^{\sigma t}e}^{j\omega t}\right)$$ (17b)

Where rotor voltage $V_{r}$ becomes zero and $R_{crow}$ shows crowbar resistance. By comparing the equations 16a, 16b and 17a,17b, we get equations 18a and 18b, which show the rotor current during unsymmetrical fault after the crowbar activates:


$$i_{dr}=\frac{L_{m}^{.}}{L_{s}}\left(\frac{\psi_{sd2}{\times e}^{jLr/R`+R_{crow\;}}}{\sqrt{{\left(R`+R_{crowbar}\right)}^{2}+{\left(L`\right)}^{2}}}\times (e^{j\omega t}-e^{\frac{t}{jL}\frac{r}{R`+R_{crow}}})\right)+\left(\frac{\psi_{sd}-\psi_{sd2})e^{jLr/R`+R_{crow\;}}}{\sqrt{{\left(R`+R_{crow}\right)}^{2}+{\left(L`\right)}^{2}}}\times (e^{j\omega t}-e^{\frac{t}{jL}\frac{r}{R`+R_{crow}}})\right)$$ (18a)

$$i_{qr}=\frac{L_{m}^{.}}{L_{s}}\left(\frac{\psi_{sq2}{\times e}^{jLr/R`+R_{crow\;}}}{\sqrt{{\left(R`+R_{crowbar}\right)}^{2}+{\left(L`\right)}^{2}}}\times (e^{j\omega t}-e^{\frac{t}{jL}\frac{r}{R`+R_{crow}}})\right)+\left(\frac{\psi_{sq}-\psi_{sq2})e^{jLr/R`+R_{crow\;}}}{\sqrt{{\left(R`+R_{crow}\right)}^{2}+{\left(L`\right)}^{2}}}\times (e^{j\omega t}-e^{\frac{t}{jL}\frac{r}{R`+R_{crow}}})\right)$$ (18b)

Here, the value of σ is low when compared with rotor damping, $e^{\sigma t}$ is intended to equal to 1.

While calculating and tunning the crowbar resistance during fault it is necessary to keeping the stator voltage below the DC- link voltage V_dc_. This voltage configuration ensures that converter can function normally even during temporary stator voltage dip. The optimal value of crowbar is calculated as R_crow_ = 0.17 p.u = 26 R_rotor._

The electromagnetic torque $T_{em}$ and stator reactive power is given as equations 19 and 20,


$$T_{em}=1.5p\frac{L_{m}}{L_{s}}(\psi_{sq}i_{dr}-\psi_{ds}i_{qr})$$ (19)

$$Q_{S}=1.5(V_{qs}i_{ds}+V_{ds}i_{qs})$$ (20)

**Fig 2:**
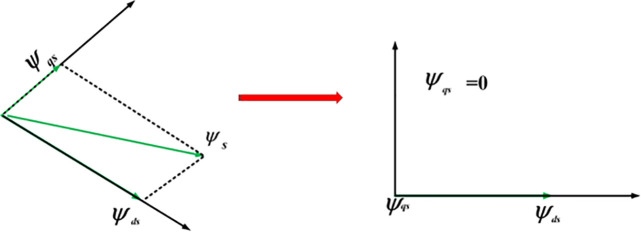
Stator flux in d-q reference frame.

The orientation of stator flux is shown in Fig 2. The stator flux is aligned with the d-axis in order to simplify and obtain reactive power control and vector control of the DFIG. $(\psi_{ds}=\psi_{s}$, $\psi_{sq}=0$) [35]:

The electromagnetic torque and reactive stator power equation can be rewritten as,


$$T_{em}=-1.5p\frac{L_{m}}{L_{s}}\psi_{s}i_{qr}$$ (21)

By putting the value of $i_{ds}$ from equation (6a), we get,


$$Q_{S}=V_{s}i_{ds}=\;1.5(\frac{V_{s}\;\psi_{ds}}{L_{m}+L_{s}}-\frac{V_{s}L_{m}}{L_{s}+L_{m}}i_{dr})$$ (22)

From equation 21, we find the reference current $i_{qr\_ref}$ shown in equation 23,


$$i_{q{r_{-}}_{ref}}=\;-0.66\frac{L_{s}}{{pL}_{m}\psi_{s}}T_{em-ref}$$ (23)

To find the d-axis reference rotor current $i_{dr\_ref}$ in order to control the reactive power, equation 22 can be rearranged as,


$$i_{dr\_ref}=\;0.66\left(\frac{\;\psi_{ds}}{L_{m}}-\frac{L_{s}+L_{m}}{V_{s}L_{m}}i_{dr}\right)Q_{S\_ref}\;$$ (24)

## 3. Proposed control scheme of RSC

**Fig 3:**
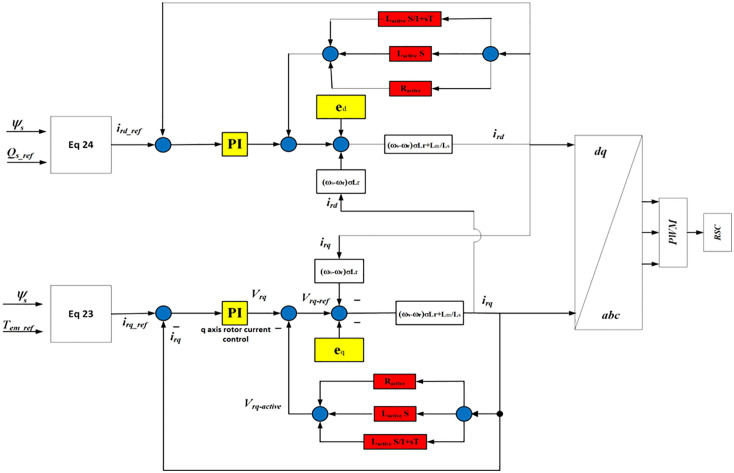
Proposed modified rotor current control scheme.


The modified rotor current control scheme, as shown in Fig 3, limits the transient current and reactive power during fault. The proposed scheme also reduces the inrush current after fault when the crowbar deactivates and RSC activates. When the fault occurs, reactive power $Q_{S}$ varies. The reactive power variation and compensation capacity that need to be adjusted during fault is achieved as follows:

$$Q_{Total}=Q_{S\_ref}-Q_{out}$$ (25)

It is observed that during the fault, the value of total reactive power $Q_{Total}$ is greater than zero, which shows that DFIG produces insufficient reactive power. In the proposed control scheme, as shown in Fig 3, when reactive power needs to be adjusted to compensate for the insufficient reactive power during a fault, a Var regulator is put in the system to meet the grid code requirement during the fault.

The enhanced crowbar structure comprises a dynamically tunable resistive-inductive branch configured to adapt based on fault severity. Unlike a fixed resistor in conventional crowbars, the proposed configuration allows the modulation of impedance via an inner current feedback loop. The feedback loop measures the rotor current magnitude and phase angle, adjusting the active resistance and inductance in real time. This dynamic control guarantees smoother activation during faults and deactivation post-fault, thus mitigating inrush currents. Fig 3 demonstrates this integration, where the crowbar operates in coordination with both rotor current feedback and outer-loop reactive power controllers.

As shown in Fig (3), the d-q axis reference currents $i_{q{r_{-}}_{ref}},\;i_{dr\_ref}$ are obtained from reactive/active power controllers from values of electromagnetic torque, stator flux, and reactive power in equations (23) and (24). The reactive power control is coordinated with the crowbar activation through a dual-loop control mechanism. During the fault, the outer loop regulates the reactive power reference based on grid code requirements, while the inner loop adjusts rotor current accordingly. When the crowbar activates (upon detection of a voltage dip), the reactive power regulator is not bypassed but remains in active control by adjusting the d-axis current reference. This guarantees that even during fault bypass, the system contributes to reactive power support. The co-ordination ensures seamless transition and avoids power quality issues by preventing excessive reactive power draw post-fault.

The value of the $i_{dr}^{*}$ is obtained by comparing the axis rotor reference current $i_{d{r-}_{ref}}$ with outer-current loop $i_{dr}$ as follows:


$$i_{dr}^{*}=i_{d{r-}_{ref}}-i_{dr}$$ (26)

Similarly,$\;i_{qr}^{*}$ is obtained by comparing the axis rotor reference current $i_{q{r-}_{ref}}$ with outer-loop current $i_{qr}$ as follow:


$$i_{qr}^{*}=i_{q{r-}_{ref}}-\;i_{qr}\;$$ (27)

$e_{d}$ and $e_{q}$ are obtained from equations (8a) and (8b), as shown in Fig (3), are the rotor back emf induced due to stator flux variation and are used to find the rotor transient currents. If the grid fault occurs the stator components varies and which changes the back emf induced the rotor and hence effects rotor dynamics and RSC. Also, rotor active impedance is established by subtracting rotor active control voltage from the output voltage of the controller as shown below:


$$V_{qr-ref}=V_{qr}-V_{qr-active}$$ (28)

$$V_{dr-ref}=V_{dr}-V_{dr-active}$$ (29)

Where,


$$V_{qr-active}=R_{active}i_{qr}+L_{active}i_{qr}+\frac{L_{active}}{\omega t}$$ (30)

$$V_{dr-active}=R_{active}i_{dr}+L_{active}i_{dr}+\frac{L_{active}}{\omega t}$$ (31)

From equations (30) and (31), the rotor active impedance is formed by the inner loop of rotor current feedback that consists of inductive and resistive values. Enhancing the crowbar design aligned with the reactive power control strategy at the outer loop and with the inclusion of active resistive and inductive terms at the inner loop can reduce the transient inrush rotor current during fault with activation and after deactivation of the crowbar.

## 4. Test system for simulation study

**Fig 4:**
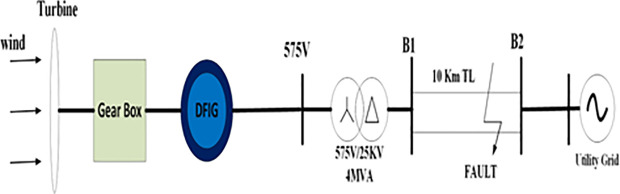
The power system with DFIG-based wind turbine under simulation study.

The test system under study is shown in Fig 4, consisting of a 2MW DFIG-based wind turbine connected to the grid station with the wind speed velocity of 12m/s. The step-up transformer of rating 0.575kV/25kV Y−Δ,4MVA supplied a power with a double circuit of a 10KM long transmission line. Two types of asymmetrical faults are applied between points B1 and B2, including single-phase faults and more severe two-phase faults. The fault occurs between $T_{fault}=2.7sec-3.2sec$. The parameters of DFIG are shown in Table 1.

**Table 1:** DFIG parameters.

Symbol	Parameters	Value
W	Rated power	2MW
*V*	Rated L-L stator voltage	690V
*A*	Rated stator current	1760A
τ	Torque at generator mode	12732 N.m
*V*	L-L Nominal rotor voltage	2070V
P	Number of Pole pairs	2
F	Stator frequency	50Hz
ω	Synchronous speed	1500 rpm
$R_{s}$	Stator resistance	2.6 mΩ
$R_{r}$	Rotor resistance	26.1 mΩ
$L_{\sigma s}$	Stator stray inductance	0.87mH
$L_{m}$	Excitation Inductance	0.25mH
$L_{\sigma r}$	Rotor stray inductance	0.783mH

The parameters in Table 1 were carefully selected to reflect realistic conditions for a 2MW Doubly Fed Induction Generator (DFIG)-based wind turbine system, based on standard industry practices and typical operating conditions. The stator resistance and rotor resistance values (2.6 mΩ and 26.1 mΩ, respectively) are chosen to model typical losses in the wind turbine’s electrical components. The stator leakage inductance (0.87 mH) and rotor leakage inductance (0.783 mH) are based on typical characteristics of the DFIG’s stator and rotor windings, ensuring the model accurately simulates fault responses under varying electrical load conditions.

The synchronous speed of 1500 rpm corresponds to a standard operational speed for wind turbines in this power range, while the nominal rotor voltage of 2070V is consistent with the voltage requirements for efficient energy conversion in grid-connected wind turbines. The magnetizing inductance (0.25 mH) reflects the transformer-like behavior of the DFIG’s stator and rotor windings, and the DC link voltage control is implemented based on the typical voltage levels necessary for reliable converter operation.

These parameter values, selected from both typical industry standards and system design constraints, ensure that the simulation accurately captures the electrical dynamics and fault behavior of a DFIG-based wind turbine in a real-world grid-connected setup.

### 4.1. Test response under asymmetrical single-phase fault

In this section, the performance of grid-connected DFIG under single-phase fault is analyzed using the MATLAB/Simulink tool. A comparative analysis of the proposed approach with conventional crowbar control and without any protection scheme is undertaken. The high amplitude of currents during fault without any protection scheme can damage the RSC, and the result shows the necessity of employing an enhanced FRT scheme. The comparative analysis of conventional crowbar protection schemes with enhanced crowbar protection shows the effectiveness of the proposed technique. Tables 2 and 3 show the quantitative comparison of the proposed scheme with conventional protection and without any protection scheme.

**Table 2:** Comparative analysis of rotor and stator parameters during single-phase fault.

	Rotor current	Stator current	q axis rotor current	d axis rotor current
Without any protection scheme	2.1 p.u	2.1 p.u	0.7 p.u	0.55 p.u
With a conventional fault protection scheme	1.1 p.u	1 p.u	0.25 p.u	0.3 p.u
With an enhanced crowbar-based scheme	0.5 p.u	0.6 p.u	0.15 p.u	0.1 p.u

**Table 3:** Comparative analysis of rotor and stator parameters during two-phase fault.

	Rotor current	Stator current	q axis rotor current	d axis rotor current
Without any protection scheme	2 p.u	2.1 p.u	1.5 p.u	1.5 p.u
With a conventional fault protection scheme	1.2 p.u	1.5 p.u	0.7 p.u	0.65 p.u
With enhanced crowbar-based scheme	1.0 p.u	1.0 p.u	0.4 p.u	0.45 p.u

Comparative Analysis of Rotor and Stator Parameters During Single-Phase Fault are given in Table 2. The enhanced crowbar-based scheme shows a significant reduction in both rotor current and stator current compared to the conventional crowbar protection. Specifically:

- **Rotor current:** The proposed method reduces the rotor current by **54.5%** (from 1.1 p.u. to 0.5 p.u.) compared to the conventional scheme.
- **Stator current:** Similarly, the stator current is reduced by **40%** (from 1.0 p.u. to 0.6 p.u.).

This reduction in current surges demonstrates a marked improvement in fault tolerance and system stability.

Comparative Analysis of Rotor and Stator Parameters During Two-Phase Fault are provided in Table 3. When comparing the proposed technique with the conventional scheme:

- **Rotor current:** The rotor current is reduced by **16.7%** (from 1.2 p.u. to 1.0 p.u.).
- **Stator current:** The stator current is reduced by **33.3%** (from 1.5 p.u. to 1.0 p.u.).
- **q-axis rotor current:** A **42.9%** reduction (from 0.7 p.u. to 0.4 p.u.) is observed in the q-axis rotor current.
- **d-axis rotor current:** The d-axis rotor current decreases by **30.8%** (from 0.65 p.u. to 0.45 p.u.).

The proposed method’s ability to limit the fault current and control reactive power leads to a more stable response under severe faults.

Comparison of Rotor and Stator Transient Currents of Different FRT Protection Strategies are given in Table 4. The proposed FRT approach outperforms several existing methods, including the DC chopper and crowbar-based schemes, in terms of fault current reduction:

**Table 4:** Comparison of rotor and stator transient currents of different FRT.

FRT approach	Rotor currents	Stator current
Crowbar circuit-based SDBS [34]	2.0 p.u	2 p.u
Time domain-based approach [35]	2.0 p.u	–
Fault Tolerant based WECS [33]	1.5 p.u	1.4 p.u
Rotor series dynamic breaking resistance protection (RSDBR) [36]	1.5 p.u	1.4 p.u
WECS based PI regulator [32]	2 p.u	2 p.u
DC chopper [37]	3.8 p.u	1.25 p.u
Cost-effective LVRT scheme [38]	2.5 p.u	2.5 p.u
DC chopper [18]	2 p.u	2 p.u
Proposed FRT approach During Two-Phase fault (crowbar-based)	1 p.u	1 p.u
Proposed FRT approach During single-phase fault (crowbar-based)	0.5 p.u	0.6 p.u

- **During two-phase fault**, the rotor current is reduced by **73.7%** (from 3.8 p.u with DC chopper to 1.0 p.u with the proposed scheme).
- **During single-phase fault**, the rotor current is reduced by **87.5%** (from 4.0 p.u with conventional methods to 0.5 p.u with enhanced crowbar protection).

These improvements demonstrate the superior ability of the proposed method to handle fault conditions and ensure system stability.

#### 4.1.1. Without any protection scheme.

**Fig 5:**
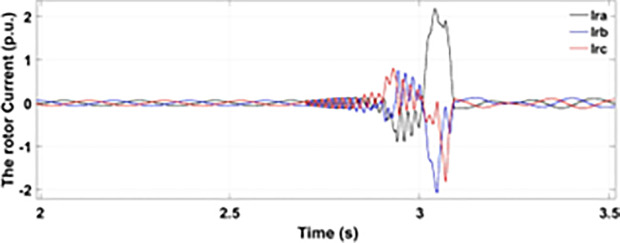
Three-phase rotor currents during single-phase fault without protection.

**Fig 6:**
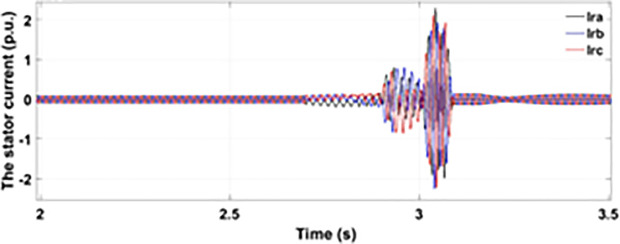
Three-phase stator current during single-phase fault without protection.

**Fig 7:**
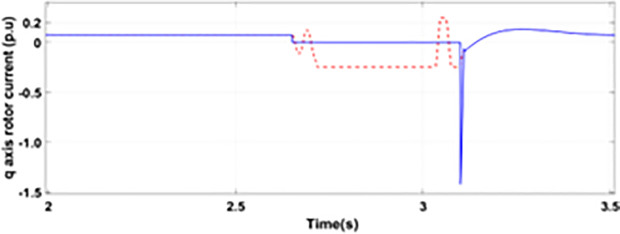
q axis rotor currents during single-phase fault without protection.

**Fig 8:**
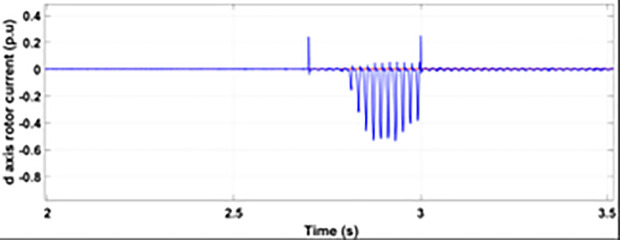
d axis rotor currents during single-phase fault without protection.

Figs 5 and 6 show a three-phase rotor and stator current, respectively, without any protective device. It can be seen that the fault current rises more than 2.0 p.u. amplitude. Meanwhile, the axis rotor current in Fig 7 has a spike of −0.7 p.u., and the d-axis current in Fig 8 has reached −0.5 p.u. Figs 5,6,7, and 8 show the importance of employing a FRT scheme. The rotor fault current of more than 2.0 p.u without any protective devices can damage the AC excitation converter (RSC) of grid-connected DFIG.

#### 4.1.2. With conventional crowbar protection scheme.

**Fig 9:**
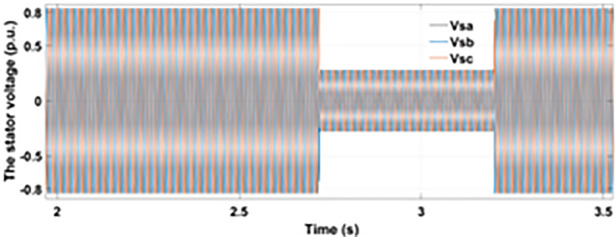
Stator voltage dip during single-phase fault.

**Fig 10:**
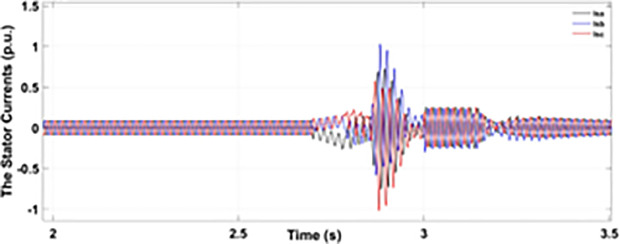
Three-phase stator current during single-phase fault with conventional protection schem.

**Fig 11:**
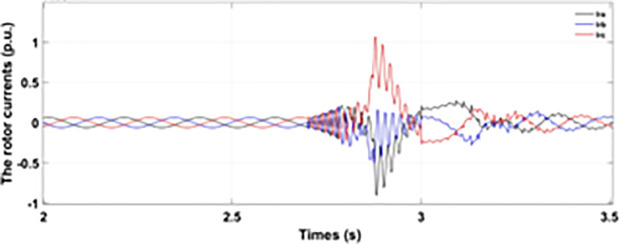
Three-phase rotor currents during single-phase fault with conventional protection scheme.

**Fig 12:**
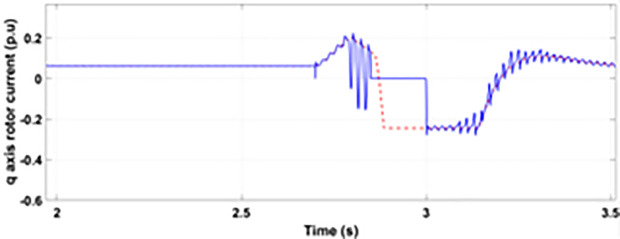
q axis rotor current during single-phase fault with conventional protection scheme.

**Fig 13:**
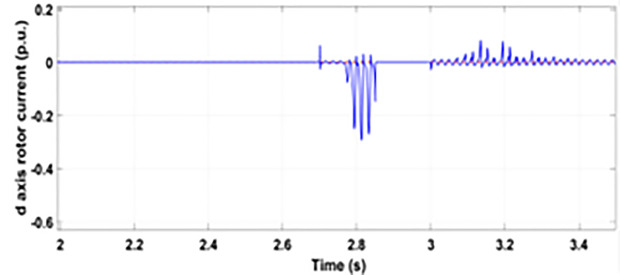
d axis rotor current during single-phase fault with conventional protection scheme.

Normally crowbar activation is designed to occur rapidly after fault detection from time 1−10 ms. In the test system under study the fault occurs between $T_{fault}=2.7-3.2s$ and crowbar activates rapidly after fault detection at time $T_{active\;}=$2.76s. Fig 9 shows the three-phase stator voltage with a voltage dip of 0.5 p.u at time T = 2.7s during a single-phase fault. Fig 10 shows a three-phase stator current with the conventional configuration of crowbar protection. It is observed that with conventional configuration, the fault surge stator current rises to 1.0 p.u. Fig 11 shows the three-phase rotor current, and it is observed that due to lack of proper current limiting technique, the rotor transient surges reached 1.0 p.u. The total span of fault is 500ms with 200ms of severe current surge that reaches 1.0 p.u. The q-axis rotor current in Fig 12 reached a surge value of 0.2 p.u and −0.25 p.u at time T = 2.85s and T = 3.0s, respectively. According to Fig 13, the amplitude of the d-axis current reached a surge of −0.3 p.u at 2.85s.

#### 4.1.3. With modified crowbar protection scheme.

**Fig 14:**
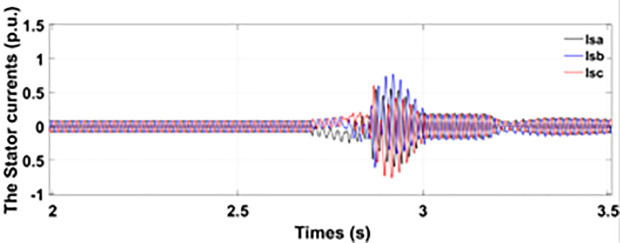
Three-phase stator currents during single-phase fault with enhanced crowbar protection scheme.

Fig 14 illustrates the behavior of the three-phase stator current during a single-phase fault event while employing the enhanced crowbar protection scheme. The modified scheme limits the fault current amplitude to 0.6 p.u, demonstrating the system’s improved fault tolerance and its ability to reduce current surges compared to conventional protection methods.

**Fig 15:**
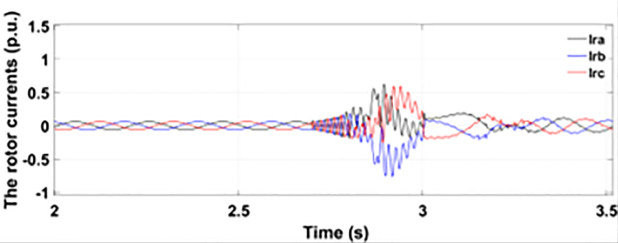
Three-phase rotor current during single-phase fault with enhanced crowbar protection scheme.

Fig 15 shows the rotor current behavior during a single-phase fault with the modified crowbar protection in place. The current is limited to 0.5 p.u, highlighting the effectiveness of the proposed technique in mitigating excessive inrush currents and stabilizing the rotor side converter (RSC) during fault conditions.

**Fig 16:**
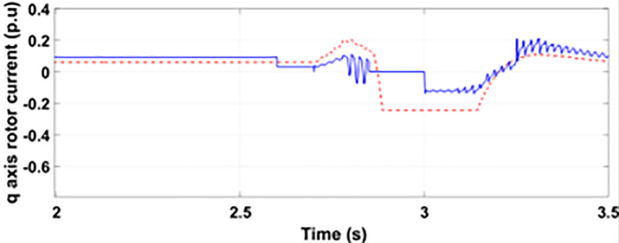
q axis rotor current during single-phase fault with enhanced crowbar protection scheme.

Fig 16 figure depicts the q-axis rotor current during a single-phase fault. The enhanced protection scheme results in a significantly lower surge, with the current reaching a maximum of −0.1 p.u. This demonstrates the improved control over transient behavior provided by the modified scheme, preventing damage to the rotor side converter.

**Fig 17:**
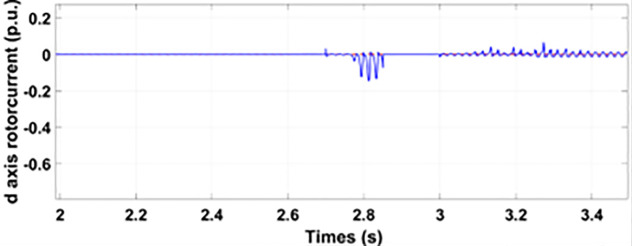
d axis rotor current during single-phase fault with enhanced crowbar protection scheme.

Fig 17 displays the d-axis rotor current behavior during a single-phase fault event with the enhanced crowbar protection scheme in action. The fault current surge is significantly reduced, with the peak amplitude reaching only 0.15 p.u. This further reinforces the capability of the modified scheme to maintain stability and minimize the risk of system failure.

### 4.2. Test response under asymmetrical two-phase fault

In this section, the comparative analysis of grid-connected DFIG without any protection, as well as a conventional and proposed scheme under severe two-phase fault, is undertaken. It is observed that without any protective devices during a severe fault, a significant increase in fault current amplitude can be observed, which can damage the rotor and RSC. In comparison, the low amplitude of fault current in rotor and stator parameters in the proposed approach shows the enhancement in FRT in terms of effectiveness and stability of system performance.

#### 4.2.1. Without any protection scheme.

**Fig 18:**
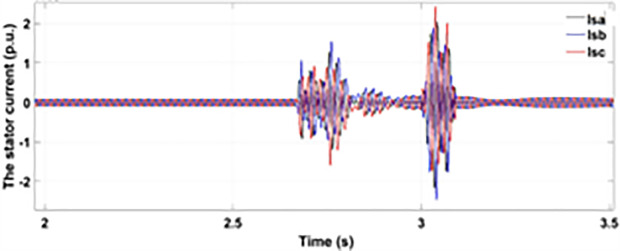
Three-phase stator currents during two-phase fault without protection.

**Fig 19:**
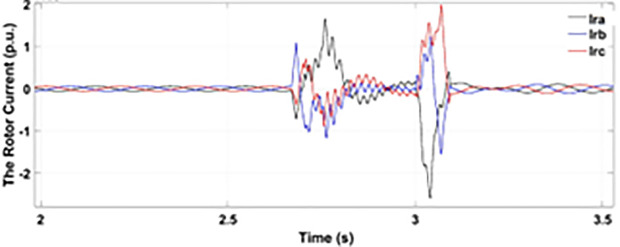
Three-phase rotor currents during two-phase fault without protection.

**Fig 20:**
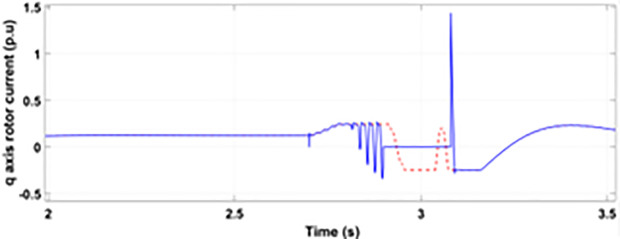
q axis rotor current during two-phase fault without protection.

**Fig 21:**
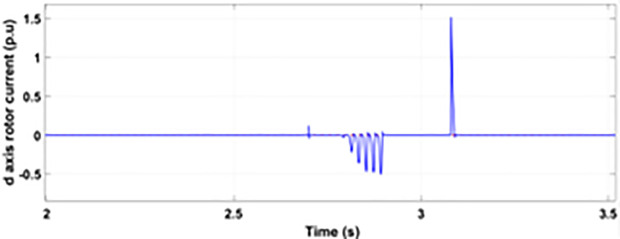
d axis rotor current during two-phase fault without protection.

When the fault amplitude of the rotor current rises above the permissible limit for a longer span, it can damage the RSC of grid-connected DFIG, and it cannot meet the grid code requirement, which can cause the shutdown and failure of the grid. From the rotor current and stator currents in Figs 18 and 19, it can be seen that a severe two-phase fault occurs with a duration of 500 ms with an amplitude of more than 2.0 p.u. The spike of 1.5 p.u is observed in the q-axis and d-axis rotor current in Figs 20 and 21, respectively. This larger duration of fault current of higher amplitude cannot meet the grid code requirement. Without any protective devices, this higher magnitude current damages the RSC of DFIG and causes system failure; therefore, an enhanced FRT is necessary to limit the fault current and avoid system failures.

#### 4.2.2. With conventional crowbar protection scheme.

**Fig 22:**
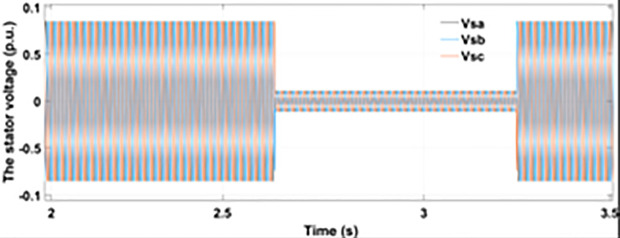
Stator voltage dip during two-phase fault.

**Fig 23:**
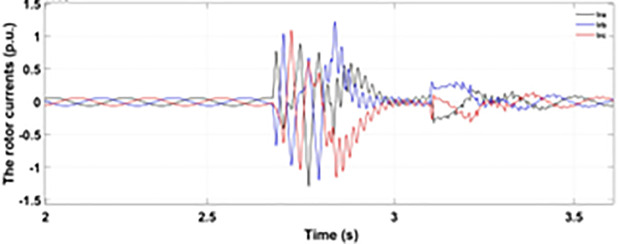
Three-phase rotor current during two-phase fault with conventional protection scheme.

**Fig 24:**
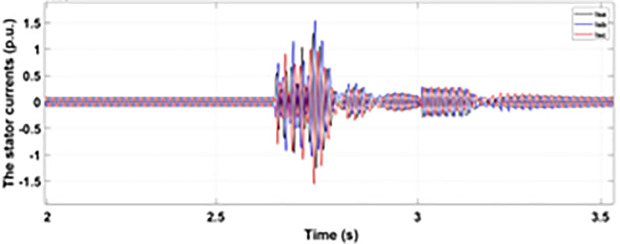
Three-phase stator current during two-phase fault with conventional protection scheme.

**Fig 25:**
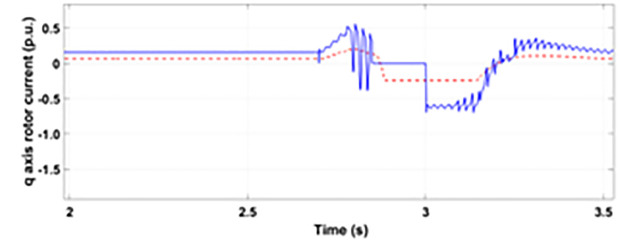
q axis rotor current during two-phase fault with conventional protection scheme.

**Fig 26:**
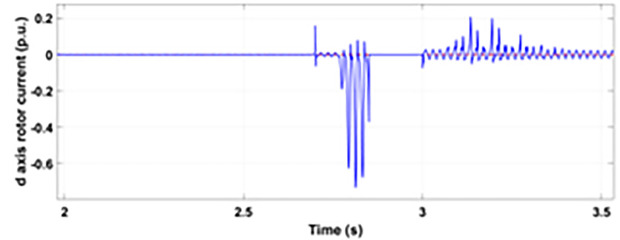
d axis rotor current during two-phase fault with conventional protection scheme.

Fig 22 shows the three-phase stator voltage with a voltage dip of 0.7 p.u at time T = 2.7s during a two-phase fault. The two-phase fault is the more severe fault that increases the amplitude of the fault for a longer span. Fig 23 shows the three-phase rotor fault current with the traditional approach. The spike of fault is 1.3 p.u and lasts for 300ms out of 500ms of total fault duration. According to Fig 24, the fault amplitude of three-phase stator currents rises to 1.5 p.u with increased transient span. Figs 25 and 26 show the q-axis and d-axis rotor current that reached fault amplitude of 0.5 p.u, −0.6 p.u and 0.15 p.u, −0.7 p.u, respectively, during two-phase fault with conventional crowbar protection.

#### 4.2.3. With modified crowbar protection scheme.

**Fig 27:**
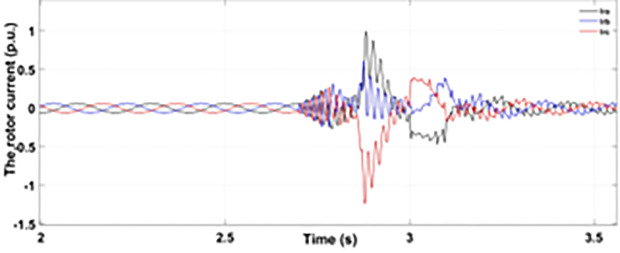
Three-phase rotor current during two-phase fault with enhanced crowbar protection scheme.

**Fig 28:**
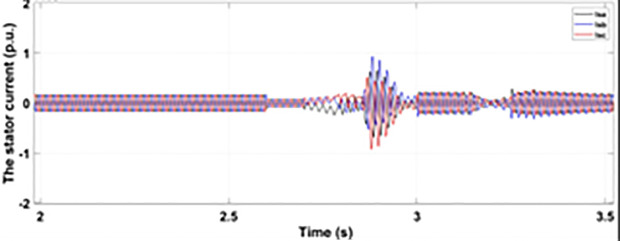
Three-phase stator current during two-phase fault with enhanced crowbar protection scheme.

**Fig 29:**
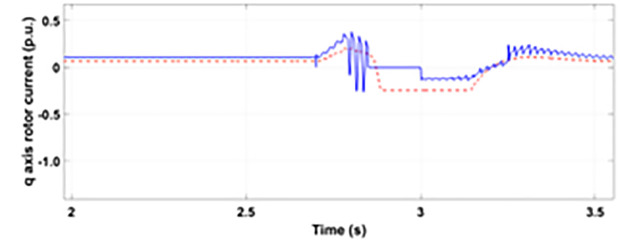
q axis rotor current during two-phase fault with enhanced crowbar protection scheme.

**Fig 30:**
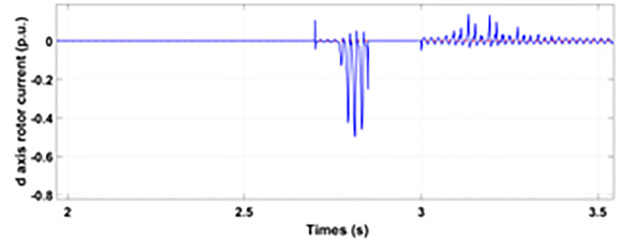
d axis rotor current during two-phase fault with enhanced crowbar protection scheme.

Fig 27 shows a three-phase rotor current with the proposed modified crowbar protection and reactive power control. By employing the modified technique, not only is the amplitude of fault reduced to 1.0 p.u, but the fault span of higher amplitude is also reduced. By employing the modified technique in Fig 28, the amplitude of the three-phase stator current is reduced to 1.0 p.u from 1.5 p.u in the conventional technique. Figs 29 and 30 show the q-axis current and d-axis current, respectively. By employing the modified technique, the transient surge is reduced to 0.4, −0.1 p.u. for the q-axis rotor current and 0.1, −0.5 for the d-axis rotor current.

### 4.3. Comparison and discussion

#### 4.3.1. Comparing different FRT techniques with proposed technique.

Table 4 shows the comparison of rotor and stator transient currents of various FRT-based techniques available in the literature, such as, DC chopper technique, crowbar circuit approach which is based on series dynamic braking resistor, fault-tolerant based approach of wind energy conversion system, cost-effective LVRT scheme and model predictive voltage control with proposed control scheme. The comparison shows the effectiveness of proposed approach and improved values of rotor and stator currents (p.u) during fault.

#### 4.3.2. Crux of the proposed strategy.

In this research, a novel rotor fault current limiting approach shows commendable effectiveness, efficiency, and stability:

- Effectiveness: The comparative analysis of conventional techniques and available literature shows the effectiveness of the proposed approach. It is observed that most of the techniques in the literature limit the rotor fault current up to 2 p.u, whereas the proposed approach limits the current to 1 p.u during severe two-phase faults. If the severity of fault is reduced to a single phase, the results of the proposed approach are improved to 0.5 p.u. The proposed scheme proved effective in protecting RSC when the severity of fault increased. Improved d and q-axis rotor current during fault further clears the insight story and shows the effectiveness of the model in handling the severity of the fault.
- Efficiency: The novel scheme shows higher efficiency by managing and optimizing the operations during single-phase faults and severe two-phase faults. The ability of the model to control reactive power facilitates voltage stabilization, which indicates the efficient operation and performance of the system.
- Stability: It can be observed from transient analysis that without any protective scheme, it can damage the RSC of DFIG and cause system instability and failure. From the comparative analysis with the literature, the ability of a proposed model to precisely control the system parameters during fault enhances the FRT capability of grid-connected DFIG-based WT and contributes to the system’s reliability and stability.
- Implementation Feasibility and Grid Compliance: The proposed scheme can be implemented in existing DFIG-based WTs through retrofitting. Since the control modifications rely on software-based enhancements (e.g., PI and Var regulators, impedance logic), present RSC hardware can accommodate the scheme with minimal physical changes. Integration with standard control platforms like dSPACE or PLCs ensures adaptability. The design supports grid code compliance by maintaining reactive power supply during faults and minimizing rotor inrush post-fault both critical for meeting LVRT (Low Voltage Ride Through) standards as per IEC 61400−21 and regional grid codes (e.g., German FGW TR3, FERC Order 661A).

#### 4.3.3. Discussion on the performance of DC choppers in stability.

While DC choppers are commonly employed in many FRT schemes to protect the DC-link capacitor and control voltage during faults, they tend to perform poorly in terms of system stability under certain fault conditions. The primary issue with DC choppers lies in their operation during severe grid disturbances.

1. **Current Limiting:** DC choppers are effective in limiting the fault current, but they tend to produce high fault current surges, especially during more severe faults, such as two-phase faults. For example, in the results of the simulation presented in Table 4, DC choppers result in a rotor current surge of up to 3.8 p.u, which is higher than the proposed crowbar-based scheme that limits the current to 1.0 p.u during a two-phase fault. This high surge in fault current is problematic, as it can severely affect the rotor side converter (RSC), increasing the risk of damage to critical components.
2. **Reactive Power Control:** DC choppers focus primarily on controlling the DC-link voltage, often neglecting the reactive power dynamics that are critical during faults. The lack of reactive power control can result in voltage instability during fault conditions, leading to prolonged recovery times and potential grid disconnection. In contrast, the enhanced crowbar-based technique proposed in this paper not only limits the fault current but also effectively controls reactive power, ensuring a more stable response during grid faults. This combination of current limiting and reactive power control significantly enhances the system’s overall stability.
3. **Inrush Current After Fault:** Another challenge with DC choppers is the high inrush current after fault recovery. When the crowbar protection deactivates and the RSC reactivates, DC choppers are slow to stabilize, leading to significant inrush currents. These inrush currents can cause damage to the RSC and compromise system reliability. The proposed enhanced crowbar scheme, however, limits these inrush currents by using active impedance control in conjunction with crowbar protection, ensuring that the system returns to normal operation with minimal current spikes.

The proposed enhanced crowbar-based FRT scheme offers a more balanced and reliable solution by addressing both current limiting and reactive power control, thus providing superior stability during fault conditions.

#### 4.3.4. Performance evaluation summary.

- **Rotor current reduction**: 87.5% during single-phase fault (from 4.0 p.u to 0.5 p.u), and 73.7% during two-phase fault (from 3.8 p.u to 1.0 p.u).
- **Stator current reduction**: 40% (from 1.0 p.u to 0.6 p.u) for single-phase fault; 33.3% (from 1.5 p.u to 1.0 p.u) for two-phase fault.
- **Reactive power support**: Controlled via d-axis current regulation in coordination with crowbar activation.
- **Grid Code Compliance**: Maintains voltage and current within acceptable limits throughout fault and post-fault periods.

## 5. Conclusion

This study presents an effective approach to enhancing the Fault Ride-Through (FRT) capability of Doubly Fed Induction Generators (DFIGs) by limiting inrush currents and controlling reactive power during faults. The proposed dynamic control technique significantly reduces fault current surges to an acceptable level, thus improving system stability during voltage dips. In comparison with conventional crowbar protection, the modified technique limits the fault current to 1 p.u during a single-phase fault and 0.5 p.u during a two-phase fault, representing reduction in fault current amplitude and duration, which greatly enhances the stability of the system. These findings have important practical implications for grid-connected wind turbines, as they allow for improved fault tolerance, ensuring that wind energy systems can continue to operate reliably even during transient grid conditions. By controlling both the fault current and reactive power, this technique enables DFIG-based wind turbines to maintain grid stability without causing shutdowns or damage to critical components.

For future research, several exciting directions can be explored. Hybrid approaches that combine DC choppers with crowbar designs could be further studied to optimize fault protection while minimizing system costs. Additionally, testing the method under extreme grid conditions—such as large-scale grid faults or prolonged voltage dips—would help assess the robustness and scalability of the proposed technique. Moreover, incorporating machine learning-based fault detection and real-time control could further enhance the adaptability and efficiency of these systems. Enhanced technique, the amplitude and surge span of fault current decrease, which shows higher system stability.

## Abbreviations

Active Impedance: Active Impedance Control
Crowbar: Crowbar Protection
DC-Link: DC-Link Voltage Control
DFIG: Doubly Fed Induction Generator
Fault Detection: Fault Detection
Faults: Asymmetrical Faults
FRT: Fault Ride-Through
Hybrid Protection: Hybrid Protection Systems
Inrush Current: Inrush Current Limiting
LVRT: Low-Voltage Ride-Through
Optimal Control: Optimal Control
Reactive Power: Reactive Power Control
RSC: Rotor Side Converter
Rotor Current: Rotor Current Limiting
Stability: Power System Stability
Surge: Fault Current Surge
Voltage Dip: Voltage Dip
WECS: Wind Energy Conversion Systems (Grid-Connected)
Wind Turbine: Wind Turbine Stability

## References

[1] Hamid C, Aziz D, Zamzoum O, El Idrissi A, Zawbaa HM, Zeinoddini‐Meymand H, et al. Intelligent control of the power generation system using DSPACE. Electronics Letters. 2024;60(9). doi: 10.1049/ell2.13193

[2] El Idrissi A, Derouich A, Mahfoud S, El Ouanjli N, Chojaa H, Chantoufi A. Bearing faults diagnosis by current envelope analysis under direct torque control based on neural networks and fuzzy logic—a comparative study. Electronics. 2024;13(16):3195. doi: 10.3390/electronics13163195

[3] Hamid C, Aziz D, Zamzoum O, El Idrissi A. Robust control system for DFIG-based WECS and energy storage in reel wind conditions. EAI Endorsed Trans Energy Web. 2024;11. doi: 10.4108/ew.4856

[4] Watil A, Chojaa H. Enhancing grid-connected PV-EV charging station performance through a real-time dynamic power management using model predictive control. Results Eng. 2024;24:103192. doi: 10.1016/j.rineng.2024.103192

[5] El Marghichi M, Loulijat A, Dangoury S, Chojaa H, Abdelaziz AY, Mossa MA, et al. Enhancing battery capacity estimation accuracy using the bald eagle search algorithm. Energy Rep. 2023;10:2710–24. doi: 10.1016/j.egyr.2023.09.082

[6] Chojaa H, Derouich A, Bourkhime Y, Chetouani E, Meghni B, Chehaidia SE, et al. Comparative study of MPPT controllers for a wind energy conversion system. In: Lecture notes on data engineering and communications technologies. Springer International Publishing; 2022. 300–10. doi: 10.1007/978-3-030-94188-8_28

[7] El Marghichi M, Dangoury S, Zahrou Y, Loulijat A, Chojaa H, Banakhr FA, et al. Improving accuracy in state of health estimation for lithium batteries using gradient-based optimization: case study in electric vehicle applications. PLoS One. 2023;18(11):e0293753. doi: 10.1371/journal.pone.0293753 37917753 PMC10621954

[8] Loulijat A, Makhad M, Hilali A, Chojaa H, El Marghichi M, Hatatah M, et al. Enhancing fault ride-through capacity of DFIG-based WPs by adaptive backstepping command using parametric estimation in non-linear forward power controller design. IEEE Access. 2024;12:58675–89. doi: 10.1109/access.2024.3381613

[9] Hadoune A, Mouradi A, Mimet A, Chojaa H, Dardabi C, Gulzar MM, et al. Optimizing direct power control of DFIG-based WECS using super-twisting algorithm under real wind profile. Front Energy Res. 2023;11. doi: 10.3389/fenrg.2023.1261902

[10] Liu X, Zhang Z, Liu Y, Liu Z, Su M, Li C, et al. Fault current unified calculation method for whole process fault ride-through of DFIG-based wind farms. IEEE Trans Smart Grid. 2024;15(1):485–503. doi: 10.1109/tsg.2023.3270702

[11] Dayo SA, Memon A, Memon ZA, Jumani TA, Abbas G, Othmen S, et al. A new approach for improving dynamic fault ride through capability of gridctied based wind turbines. Sci Rep. 2025;15(1):6144. doi: 10.1038/s41598-025-89396-0 39979423 PMC11842805

[12] Musarrat MN, Fekih A, Rahman MdA, Islam MR, Muttaqi KM. An event triggered sliding mode control-based fault ride-through scheme to improve the transient stability of wind energy systems. IEEE Trans on Ind Applicat. 2023:1–11. doi: 10.1109/tia.2023.3328851

[13] Raghavendran CR, Preetha Roselyn J, Devaraj D. Development and performance analysis of intelligent fault ride through control scheme in the dynamic behaviour of grid connected DFIG based wind systems. Energy Rep. 2020;6:2560–76. doi: 10.1016/j.egyr.2020.07.015

[14] Talani RA, Kaloi GS, Ali A, Bijarani MA, Abbas G, Hatatah M, et al. Dynamic performance analysis and fault ride-through enhancement by a modified fault current protection scheme of a grid-connected doubly fed induction generator. Machines. 2025;13(2):110. doi: 10.3390/machines13020110

[15] Chang Y, Kocar I, Farantatos E, Haddadi A, Patel M. Short-circuit modeling of DFIG-based WTG in sequence domain considering various fault- ride-through requirements and solutions. IEEE Trans Power Delivery. 2023;38(3):2088–100. doi: 10.1109/tpwrd.2023.3235985

[16] Rasool S, Muttaqi KM, Sutanto D. A novel fault ride-through capability improvement scheme for the hybrid offshore wind-wave energy conversion systems. Electric Power Syst Res. 2023;217:109166. doi: 10.1016/j.epsr.2023.109166

[17] Ansari AA, Dyanmina G. Real-time implementation of the fuzzy logic controlled parallel protection technique to enhance the DFIG system’s FRT capability. Scientia Iranica. 2023;0(0):0–0. doi: 10.24200/sci.2023.61209.7200

[18] Ansari AA, Dyanamina G. MATLAB Simulation of FRT Techniques for DFIG-based Wind Farms. In: 2021 International conference on control, automation, power and signal processing (CAPS). 2021. 1–6. doi: 10.1109/caps52117.2021.9730674

[19] Omidi A, Kalantar M. Improved fault ride through strategy of doubly fed induction generator based wind turbine using model predictive control. In: 7th Iran wind energy conference (IWEC2021). 2021. 1–6. doi: 10.1109/iwec52400.2021.9467000

[20] Loulijat A, El Marghichi M, Makhad M, Ettalabi N. Crowbar with a parallel RpLp configuration using PI controller to solve the problem of DFIG-wind farm stability during a symmetrical fault. Int J Intell Eng Syst. 2023;16(6):799–812. doi: 10.22266/ijies2023.1231.66

[21] Naderi SB, Negnevitsky M, Muttaqi KM. A modified DC chopper for limiting the fault current and controlling the DC-link voltage to enhance fault ride-through capability of doubly-fed induction-generator-based wind turbine. IEEE Trans on Ind Applicat. 2019;55(2):2021–32. doi: 10.1109/tia.2018.2877400

[22] Nguyen M-D, Kim T-S, Shin K-H, Jang G-H, Choi J-Y. Fast prediction of characteristics in wound rotor synchronous condenser using subdomain modeling. Mathematics. 2024;12(22):3526. doi: 10.3390/math12223526

[23] Onishi K, Li Y, Koiwa K, Liu F, Zanma T, Liu K-Z. Analysis on the operation of crowbar in doubly fed induction generators. Electric Power Syst Res. 2023;215:108950. doi: 10.1016/j.epsr.2022.108950

[24] Bekiroglu E, Yazar MD. Improving fault ride through capability of DFIG with fuzzy logic controlled crowbar protection. In: 2022 11th International conference on renewable energy research and application (ICRERA). 2022. 374–8. doi: 10.1109/icrera55966.2022.9922804

[25] Xiao F, Xia Y, Zhang K, Zhang Z, Yin X. Fault characteristics analysis of DFIGWT in whole LVRT process considering control strategy switching between RSC and Crowbar. Int J Electrical Power Energy Syst. 2023;145:108615. doi: 10.1016/j.ijepes.2022.108615

[26] Reddy K, Saha AK. A Heuristic approach to optimal crowbar setting and low voltage ride through of a doubly fed induction generator. Energies. 2022;15(24):9307. doi: 10.3390/en15249307

[27] Lin S, Yao W, Xiong Y, Shi Z, Zhao Y, Ai X, et al. Three-stage dynamic equivalent modeling approach for wind farm using accurate crowbar status identification and voltage differences among wind turbines. Electric Power Syst Res. 2024;228:110091. doi: 10.1016/j.epsr.2023.110091

[28] Li J, Qiu Q, Zhan M. An improved crowbar control circuit of DFIG during LVRT. In: 2021 IEEE 4th International Electrical and Energy Conference (CIEEC). 2021. 1–6. doi: 10.1109/cieec50170.2021.9510598

[29] Goni R, Salunke M, Raju AB. Modeling and simulation of crowbar protection technique for doubly fed induction generator under symmetrical fault condition. In: 2024 3rd International Conference for Innovation in Technology (INOCON). 2024. 1–8. doi: 10.1109/inocon60754.2024.10511624

[30] Swain S, Ray PK. Short circuit fault analysis in a grid connected DFIG based wind energy system with active crowbar protection circuit for ridethrough capability and power quality improvement. Int J Electrical Power Energy Syst. 2017;84:64–75. doi: 10.1016/j.ijepes.2016.05.006

[31] Cortajarena JA, Barambones O, Alkorta P, Cortajarena J. Grid frequency and amplitude control using DFIG wind turbines in a smart grid. Mathematics. 2021;9(2):143. doi: 10.3390/math9020143

[32] Krause PC. Analysis of electric machinery. 2nd ed. New York: McGraw-Hill; 2002.

[33] Döşoğlu MK. Crowbar hardware design enhancement for fault ride through capability in doubly fed induction generator-based wind turbines. ISA Trans. 2020;104:321–8. doi: 10.1016/j.isatra.2020.05.024 32423617

[34] Rahimi M, Azizi A. Transient behavior representation, contribution to fault current assessment, and transient response improvement in DFIG-based wind turbines assisted with crowbar hardware. Int Trans Electr Energ Syst. 2018;29(1):e2698. doi: 10.1002/etep.2698

[35] Jabal Laafou A, Ait Madi A, Addaim A. Dynamic control of DFIG used in wind power production, based on PI regulator. In: IEEE 2nd International conference on electronics, control, optimization and computer science. 2020.

[36] Ali MAS, Mehmood KK, Baloch S, Kim C-H. Modified rotor-side converter control design for improving the LVRT capability of a DFIG-based WECS. Electric Power Syst Res. 2020;186:106403. doi: 10.1016/j.epsr.2020.106403

[37] Okedu KE, Muyeen SM, Takahashi R, Tamura J. Wind farms fault ride through using DFIG with new protection scheme. IEEE Trans Sustain Energy. 2012;3(2):242–54. doi: 10.1109/tste.2011.2175756

[38] Zou X, Zhu D, Hu J, Zhou S, Kang Y. Mechanism analysis of the required rotor current and voltage for DFIG-based WTs to ride-through severe symmetrical grid faults. IEEE Trans Power Electron. 2018;33(9):7300–4. doi: 10.1109/tpel.2018.2799218

[39] Ansari AA, Dyanamina G. Fault ride-through operation analysis of doubly fed induction generator-based wind energy conversion systems: a comparative review. Energies. 2022;15(21):8026. doi: 10.3390/en15218026

[40] Pannell G, Zahawi B, Atkinson DJ, Missailidis P. Evaluation of the performance of a DC-link brake chopper as a DFIG low-voltage fault-ride-through device. IEEE Trans Energy Convers. 2013;28(3):535–42. doi: 10.1109/tec.2013.2261301

[41] Al-Barashi MM, Ibrahim DK, El-Zahab EE-DA. Evaluating the energy system in Yemen. J Elect Eng. 2016;16(1):338–42.

[42] Al-Barashi MM, Ibrahim DK, El-Zahab EE-DA. Evaluating connecting Al-Mukha new wind farm to yemen power system. Int J Electr Energy. 2015;3(2). doi: 10.12720/ijoee.3.2.57-67

